# Research Advances and Future Perspectives of Point-of-Care Detection Technologies and Biosensors for Mosquito-Borne Viruses

**DOI:** 10.3390/bios16090474

**Published:** 2026-08-29

**Authors:** Erkang Bian, Ruohang Wang, Kun Yin, Xiong Ding

**Affiliations:** 1 Key Laboratory of Environmental Medicine and Engineering, Ministry of Education, Department of Nutrition and Food Hygiene, School of Public Health, Southeast University, Nanjing 210009, China; 2 School of Global Health, Chinese Center for Tropical Diseases Research, Shanghai Jiao Tong University School of Medicine, Shanghai 200025, China; 3 Southeast University Suzhou Research Institute, Suzhou 215123, China

**Keywords:** mosquito-borne viruses, point-of-care testing, biosensors, nucleic acid amplification, CRISPR diagnostics, microfluidics biosensors, lateral-flow assays, electrochemical biosensors

## Abstract

Mosquito-borne viruses, including dengue, Zika and chikungunya viruses, place a substantial burden on diagnostic services, especially where molecular laboratories are inaccessible or slow to return results. Point-of-care biosensors could reduce turnaround times and bring testing closer to patients in primary care, outbreak response, and field settings. This review critically examines nucleic acid amplification, CRISPR-assisted assays, lateral-flow platforms, microfluidic systems, electrochemical and optical biosensors, paper-based devices, and smartphone-enabled readouts. These technologies are evaluated in terms of sample preparation, analytical sensitivity and specificity, matrix interference, multiplexing, workflow integration, cost, and clinical validation. Overall, nucleic-acid-amplification and CRISPR-assisted platforms often achieve low reported detection limits under controlled conditions; lateral-flow and paper-based devices offer relatively simple and minimally instrumented workflows; and microfluidic, electrochemical, and smartphone-enabled systems support increasing levels of workflow integration, quantitative readout, and connectivity. However, few platforms currently integrate these advantages into a fully integrated and clinically validated “sample-to-result” workflow. Due to sample heterogeneity, viral strains, reference methods, assay conditions, and disparities in reporting practices, conducting meaningful cross-study comparisons remains challenging. Limited comparisons and insufficient prospective clinical and field validation further restrict the assessment of practical diagnostic utility. Therefore, strong analytical performance alone should not be interpreted as evidence of clinical validity. Priority directions include unified definitions of performance and reporting units, standardized validation protocols and external quality assessment, prospective multi-site evaluation using representative populations and specimens, and earlier consideration of manufacturing scalability, reagent stability, quality systems, and applicable regulatory requirements. Future platforms should integrate simplified sample preparation, multiplex detection, objective digital or AI-assisted interpretation, and secure connectivity while demonstrating measurable benefits for patient management and outbreak surveillance.

## 1. Introduction

Mosquito-borne viruses (MBVs) continue to be a serious public health issue. Their global range has grown in tandem with urbanization, population migration, international trade, and climate change. More than 5 billion individuals are thought to be at risk of contracting one or more of the MBVs that have been documented in more than 100 tropical and subtropical nations [1,2,3,4].

Broadly effective vaccines and virus-specific antiviral drugs remain unavailable for many MBV infections. This situation increases the need for rapid, sensitive, and field-deployable diagnostic assays and biosensing platforms for surveillance, early case detection, and outbreak response [5].

The clinical diagnosis of MBV infections is challenging because many infections present with overlapping and nonspecific manifestations, including fever, rash, arthralgia, myalgia, headache, and malaise. In regions where dengue virus (DENV), Zika virus (ZIKV), chikungunya virus (CHIKV), Japanese encephalitis virus (JEV), West Nile virus (WNV), and other arboviruses co-circulate, clinical manifestations alone are insufficient for reliable etiological diagnosis. Misclassification can affect patient management, pregnancy counseling, vector-control decisions, and outbreak reporting. Pathogen-specific laboratory assays and biosensor-based detection systems are therefore important for confirming acute infection, differentiating among co-circulating viruses, and guiding public-health interventions.

MBV detection has evolved from virus isolation and culture [2] to antigen–antibody assays [6], reverse-transcription polymerase chain reaction (RT-PCR) [7] and more recently portable biosensing technologies. Conventional laboratory methods have substantially improved diagnostic accuracy, but many depend on specialized equipment, trained personnel, controlled workflows, and cold-chain storage or stable reagents. Turnaround time, cost, and limited access further restrict their use outside centralized laboratories. Diagnosis is additionally complicated by the co-circulation of MBVs that produce similar clinical manifestations, increasing the need for biosensors with high analytical specificity, multiplexing capacity, and clear differential diagnostic value [8]. Because many outbreaks occur in areas with limited laboratory capacity, simple, portable, and robust biosensing platforms may provide practical alternatives or complements to conventional testing [9]. 

Serological diagnosis is particularly challenging for flaviviruses. Because DENV, ZIKV, JEV, WNV, and yellow fever virus share antigenic epitopes, the antibodies elicited by these viruses may cross-react. Such cross-reactivity can reduce the specificity of IgM- and IgG-based assays and immunosensors, especially in populations with a history of flavivirus infection or vaccination. Neutralization assays may offer greater specificity, but they are labor-intensive, slow, and generally confined to specialized laboratories. Serological and antibody-biosensor findings must therefore be interpreted with reference to the stage of infection, vaccination history, and local flavivirus epidemiology, as well as—where available—molecular or antigen-based evidence of acute infection.

Against this background, biosensors have emerged as promising tools for translating pathogen recognition into rapid and potentially field-deployable diagnostic readouts. A biosensor typically combines a biological recognition element such as an antibody, aptamer, nucleic acid probe, or CRISPR-associated system with a transducer that converts a target-binding, amplification, or cleavage event into a measurable optical, electrochemical, colorimetric, or other signal [10]. For MBV detection, biosensors can target viral RNA, viral antigens, or host antibodies. These biosensing systems can also be integrated with isothermal nucleic acid amplification, lateral-flow strips, paper-based devices, microfluidic platforms, and smartphone-assisted readers [11]. Such integration may reduce assay time, reagent consumption, and dependence on centralized instrumentation while improving portability and readout objectivity. Biosensors should nevertheless be distinguished from stand-alone amplification or laboratory assays: amplification-assisted methods constitute integrated biosensors only when target recognition is coupled to a defined signal-transduction and readout component. A low analytical limit of detection alone does not ensure the clinical validity or utility of an MBV biosensor. Sample preparation, matrix effects, cross-reactivity, signal stability, multiplexing capability, manufacturing reproducibility, user-dependent interpretation, and validation using representative clinical specimens remain critical determinants of real-world biosensor performance [12]. A related concern is that many reported MBV biosensors have been evaluated mainly with synthetic targets, cultured viruses, or small convenience sample sets. Their adoption in routine practice will require prospective clinical studies, standardized reference materials, external quality assessment, and direct comparison with established laboratory methods.

By shortening the interval between sample collection and result reporting, point-of-care testing has altered some aspects of clinical decision-making [13]. The World Health Organization (WHO) developed the ASSURED criteria to define the desired features of point-of-care diagnostics for resource-limited settings: Affordable, Sensitive, Specific, User-friendly, Rapid and robust, Equipment-free, and Deliverable [14]. This framework is useful for determining whether an MBV biosensor provides adequate analytical performance while remaining operationally feasible. ASSURED was later expanded to REASSURED through the inclusion of Real-time connectivity, Ease of specimen collection, and broader accessibility [15]. Field-deployable biosensors should thus be judged on more than analytical sensitivity and specificity. Specimen requirements, device stability, connectivity, ease of use, cost, and compatibility with decentralized testing workflows are equally relevant.

**Figure 1:**
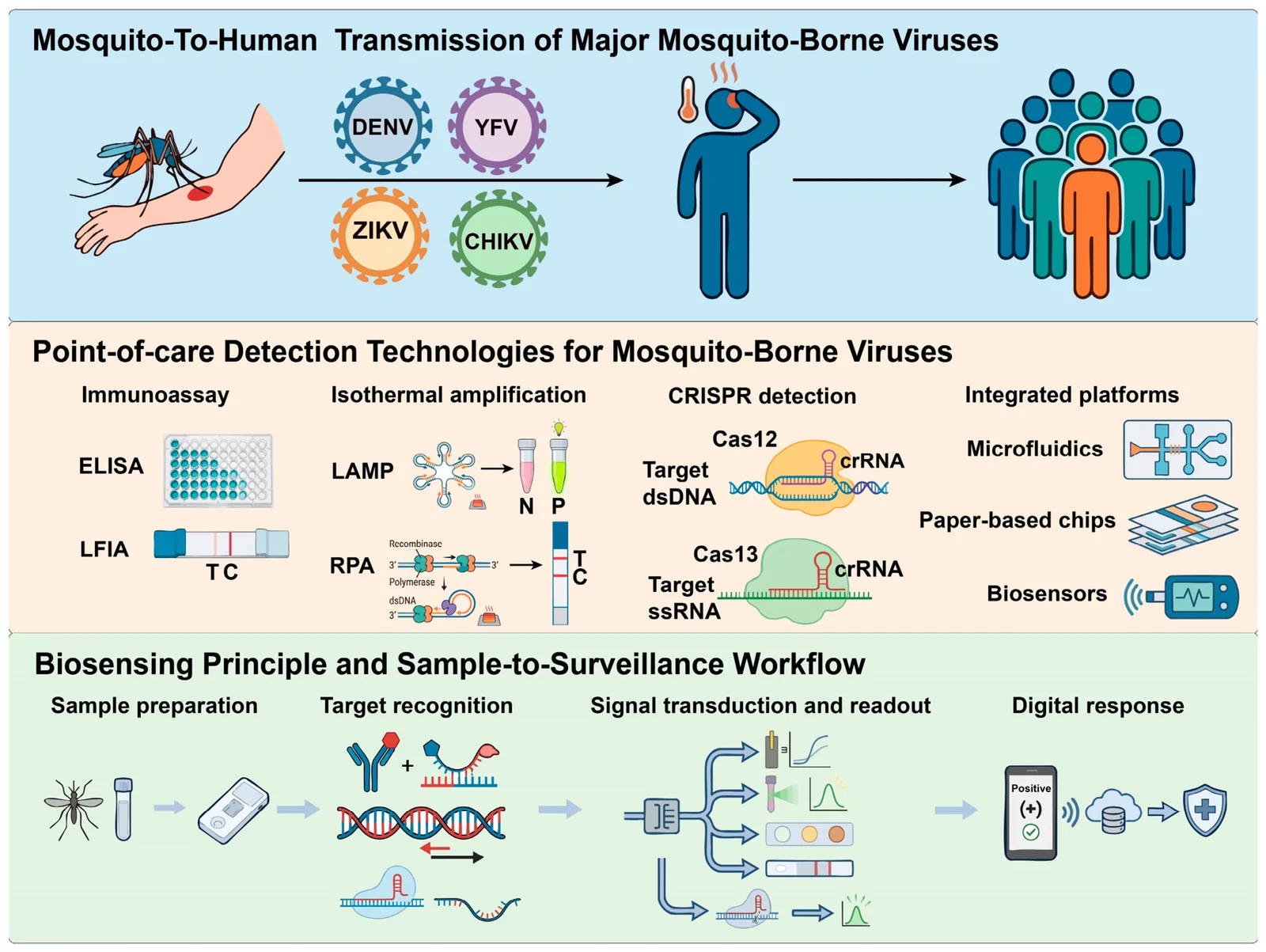
Overview of point-of-care detection technologies and biosensors for mosquito-borne virus (MBV) detection.

Several recent reviews have examined arbovirus diagnostics or technologies developed specifically for dengue detection [16,17]. The present review differs in three respects. First, it focuses on the biosensing layer of point-of-care detection rather than on assay chemistry alone, with explicit attention to signal transduction, device integration, and readout objectivity. Second, it separates the analytical validity of MBV biosensors from their clinical validity and clinical utility and identifies where each biosensing platform currently lies along this translational continuum. Third, it examines biosensor standardization, quality control, manufacturing reproducibility, and regulatory pathways as prerequisites for translation rather than as post hoc considerations. Figure 1 shows the overview of point-of-care MBV detection technologies and biosensors mentioned in this review.

## 2. Virological Targets, Diagnostic Windows, and Biosensor Design Considerations

The performance of MBV diagnostics depends on the selected target, sampling time, specimen matrix, and analytical workflow. Viral RNA, antigens, infectious virus, IgM, and IgG appear at different stages of infection [18,19]. In this review, diagnostic windows are expressed as days post-symptom onset (DPO), with symptom onset defined as DPO 0, to allow more precise comparison across targets. Therefore, even a highly sensitive biosensor may show poor clinical sensitivity if the target is absent or present at a low concentration during the intended diagnostic window.

Table 1 shows the virological targets and diagnostic windows for MBV detection. Viral RNA and antigens are generally preferred during acute infection, whereas IgM and IgG are more informative after the host immune response develops. Biosensor design should align the diagnostic target and window with the biorecognition element, signal-transduction mechanism, specimen type, and intended use. Field-deployable platforms must also account for sample degradation, matrix interference, simplified processing, and limited storage conditions [20]. Validation should therefore use clinically representative specimens rather than only purified or spiked targets.

### 2.1. Viral RNA

Viral RNA is the preferred molecular target for the early diagnosis of acute MBV infection. For many MBVs, including DENV, ZIKV, and CHIKV, viral RNA is most reliably detected during the early febrile phase, generally within the first several days after symptom onset [21,22]. Conventional nucleic acid amplification tests, including reverse-transcription quantitative polymerase chain reaction (RT-qPCR), reverse-transcription loop-mediated isothermal amplification (RT-LAMP), and reverse-transcription recombinase polymerase amplification (RT-RPA), are therefore most useful when patients present early or when surveillance is performed using freshly collected vector specimens [23].

These amplification methods are not automatically biosensors. They become components of amplification-assisted biosensors only when sequence-specific recognition is connected to a measurable fluorescent, colorimetric, electrochemical, or lateral-flow output. Similarly, CRISPR-based biosensors combine Cas-mediated target recognition with reporter-based signal generation. Integrated platforms may further combine extraction, amplification, recognition, and readout.

RNA detection is strongly affected by sampling time, viral load, degradation, extraction efficiency, and matrix inhibition. Thus, a negative result does not necessarily exclude infection, especially after the viremic phase. Internal extraction and amplification controls are essential [24]. Biosensor validation should additionally evaluate matrix interference, specificity against related viruses, signal stability, reproducibility, and clinical sensitivity.

### 2.2. Viral Antigen

Viral antigens are suitable targets for acute-phase diagnosis. DENV NS1 is the most established example and can be detected in serum or plasma early in infection [25,26]. Conventional NS1 ELISAs and rapid tests are widely used because they do not require nucleic acid extraction [27].

Antigen biosensors or immunosensors use antibodies or other affinity reagents to capture the target and convert binding into an optical, colorimetric, electrochemical, or digital signal. Lateral-flow biosensors are especially suitable for decentralized testing, while electrochemical and optical systems may provide quantitative results.

NS1 detection can vary with serotype, sampling time, primary or secondary infection, and specimen matrix. In secondary DENV infection, immune-complex formation may reduce the amount of free NS1 available for detection [28]. Cross-reactivity and nonspecific adsorption must also be considered. Performance obtained with recombinant antigen in buffer should not be directly extrapolated to clinical specimens. Combining antigen detection with RNA or antibody testing may broaden the diagnostic window [29].

### 2.3. IgM Antibodies

IgM usually becomes detectable several days after symptom onset and may persist for weeks or months [30]. IgM-capture ELISAs, rapid immunochromatographic assays, and serological biosensors are therefore useful when viral RNA or antigen is no longer detectable [31].

IgM biosensors generally use immobilized viral antigens or antibody-capture reagents, with colorimetric, fluorescent, electrochemical, or lateral-flow readouts. However, IgM is often absent during very early infection. Flavivirus IgM detection is also affected by cross-reactivity among DENV, ZIKV, JEV, WNV, and yellow fever virus, especially in regions with co-circulation or vaccination [32].

Specificity may be improved using virus-specific epitopes, recombinant antigens, or multiplex biosensing. Nevertheless, multiplexing does not fully eliminate cross-reactivity, and neutralization assays may still be required for confirmation [33]. Clinical validation should include specimens from patients with related infections and previous flavivirus exposure or vaccination.

### 2.4. IgG Antibodies

IgG is mainly used to assess previous exposure, immune status, or secondary infection. In primary infection, IgG usually appears after IgM, whereas secondary flavivirus infection may produce a rapid IgG response [34].

IgG biosensors use immobilized antigens coupled to optical, electrochemical, or colorimetric transducers. Simultaneous IgM and IgG detection may improve interpretation, but results remain dependent on sampling time, previous exposure, vaccination, and local epidemiology [35].

A single positive IgG result cannot reliably distinguish current infection from previous infection or vaccination. Cross-reactive flavivirus antibodies may also reduce biosensor specificity. Paired sera, seroconversion, changes in antibody titer, or neutralization assays may therefore be required [32]. IgG biosensors are consequently more appropriate for serosurveillance or multi-marker platforms than for stand-alone confirmation of acute infection.

### 2.5. Infectious Virus

Virus isolation, plaque assays, focus-forming assays, and TCID_50_ assays detect replication-competent virus and provide information that RNA, antigen, and antibody biosensors cannot directly provide [36]. A positive RNA or antigen result indicates the presence of a viral component but does not necessarily demonstrate infectivity.

Infectious-virus detection is limited by a short recovery window and strict requirements for specimen handling, biosafety facilities, cell culture, and trained personnel [37]. These methods are therefore mainly used for reference confirmation, virus characterization, and biosensor validation rather than routine field diagnosis.

Cell-based biosensors may offer functional detection of viral infection, but their specificity, robustness, response time, and biosafety require further validation.

### 2.6. Viral RNA in Mosquito Specimens

Viral RNA is the main target for testing individual mosquitoes and mosquito pools. RT-qPCR remains the reference method, while RT-LAMP, RT-RPA, portable PCR, sequencing, and nucleic acid biosensors are being investigated for field surveillance [38]. Mosquito testing may provide early evidence of local viral circulation before human cases increase [39].

Stand-alone amplification or sequencing methods should be distinguished from biosensors. Amplification-assisted and CRISPR-based biosensors connect sequence recognition to fluorescent, colorimetric, electrochemical, or lateral-flow outputs. Microfluidic platforms may further integrate mosquito homogenization, RNA extraction, amplification, and signal readout.

Mosquito tissues, blood meals, environmental contaminants, pool size, and storage conditions can affect target recovery and biosensor signals. Validation should therefore use infected or field-collected mosquitoes at realistic pool sizes rather than purified RNA alone [40]. Process controls should monitor homogenization, extraction, amplification, and signal generation.

Biosensor-based mosquito surveillance may complement established molecular testing by enabling portable detection and rapid data transmission. However, viral RNA detected in an engorged mosquito may originate from a recent blood meal and does not necessarily confirm an established mosquito infection.

## 3. Specimen Types, Matrix Effects, and Biosensor Integration for Mosquito-Borne Virus Detection

Specimen selection strongly affects MBV detection because viral RNA, antigens, and antibodies vary with infection stage and sample type. Serum, whole blood, urine, saliva, cerebrospinal fluid (CSF), and mosquito homogenates differ in target abundance, inhibitors, background components, and storage requirements [21,41]. These matrix effects may influence target recovery, biorecognition, signal transduction, capillary flow, electrode fouling, and optical background. Table 2 summarizes the methodological considerations associated with each specimen type for MBV detection.

Biosensors validated only with purified or spiked targets may not perform similarly in clinical or field specimens [42]. Matrix-specific validation should therefore assess the complete specimen-to-result workflow, including collection, preparation, recognition, signal generation, and readout. RT-qPCR, RT-LAMP, ELISA, and sequencing should be described as conventional methods unless they are integrated with biorecognition, signal transduction, and measurable output.

### 3.1. Human Clinical Specimens

#### 3.1.1. Serum and Plasma

Viral RNA, DENV NS1 antigen, IgM, and IgG can be commonly detected in both serum and plasma using conventional assays as well as nucleic acid, antigen, and serological biosensors [25,31]. Nevertheless, they are still problematic in field applications, as the process of taking, handling, storing, and processing blood samples is challenging, and hemolysis, lipemia, anticoagulants, and protein adsorption can affect the biosensor performance. Portability can be enhanced with integrated plasma separation and/or microfluidic processing, though these options need to be validated using existing lab processes [43].

#### 3.1.2. Whole Blood

Whole blood can be used for finger-prick collection and point-of-care testing without needing immediate centrifugation and can be used with lateral-flow, paper-based, or microfluidic and electrochemical biosensors [15]. The complexity of this matrix is still significant as blood cells, hemoglobin, proteins, and anticoagulants can inhibit amplification, change the flow of capillaries or interfere with the optical and electrochemical signals. Devices may then need to be designed for built-in lysis, removal of inhibition, or plasma separation, and validation may involve evaluating hematocrit, target recovery, matrix inhibition and performance of the process controls [44].

#### 3.1.3. Dried Blood Spots and Filter-Paper-Based Specimens

Dried blood spots, such as on FTA cards or filter paper, have advantages for transporting and storing in resource-limited areas [45]. The format can also help minimize the need for cold chain maintenance and facilitate a retrospective molecular or serological analysis, making it particularly appropriate for surveillance, seroepidemiological studies and outburst investigations in remote regions [46].

There are important limitations to consider: the amount of specimen that can be obtained from dried blood spots is usually small, and yields of nucleic acid or antibody may be lower than in liquid samples. Other factors that can affect sensitivity include extraction efficiency, elution volume, drying conditions, humidity, and storage time, necessitating matrix-specific validation when these specimens are used to detect early-stage infections and/or cases with low viremia [47].

#### 3.1.4. Urine

Urine has predominantly been used for Zika virus testing since ZIKV RNA can persist in urine for a longer time in some patients compared to serum [48]. Urine is a useful field- and community-based sample for operationally simple, non-invasive collection [49].

The viral load and composition of urine are highly variable and can be processed by molecular methods and nucleic acid biosensors. RNA is susceptible to instability or interference with biosensor signals from salts, nucleases, pH changes and cellular materials. Concentration or extraction may increase sensitivity but both increase workflow; thus, urine is a better choice for a complementary specimen [50].

#### 3.1.5. Saliva

Another non-invasive specimen that is analyzed for ZIKV, dengue virus and chikungunya virus is saliva. Collection is simple and can be considered for repeated collections, pediatric testing, and for decentralized screening and surveillance, making saliva-based methods appealing [51].

Although collection is simple, analytical challenges exist due to the interference of mucins, enzymes, microorganisms, food residues and fluctuating viscosity which can cause amplification to fail or disrupt biosensor readout. The viral load may also be lower than in the blood. Standardized collection, stabilization and preparation procedures, and appropriate process controls are therefore essential for saliva-based biosensors, as do clinical specimen and artificial or spiked saliva validation [52].

#### 3.1.6. Cerebrospinal Fluid

Clinical importance of CSF is seen in cases of mosquito-borne viruses producing neurological disease, such as Japanese encephalitis virus, West Nile virus and some cases of Zika virus infection. It is often useful to detect CSF IgM since viral RNA may be absent or only present for a short time at the time of neurological signs [53].

The CSF is suitable for both nucleic acid and antibody biosensors, and it is necessary to have high analytical sensitivity and a low background level. In addition, CSF differs significantly from serum and is only accessible in an invasive fashion, so the clinical validation and matrix specific cut-offs are therefore required [54].

#### 3.1.7. Semen

Semen is important for Zika virus as the virus was found to persist for a longer period in this fluid, and sexual transmission has been epidemiologically reported. Testing may then be warranted in specific clinical and/or public health contexts such as counseling following possible exposure to ZIKV [55].

It is very viscous and contains cells, proteins and enzymes, making extraction as well as biosensing difficult, occasionally requiring standardized pretreatment. Viral RNA should not be considered as a diagnostic specimen but as a virus-specific specimen, meaning that the detection of its RNA does not imply the presence of infectious virus or the likelihood of an ongoing transmission risk [56].

### 3.2. Vector Specimens

#### 3.2.1. Individual Mosquitoes

Individual mosquito testing provides detailed information about infection status, mosquito species, and the spatial distribution of viral circulation. This method is valuable for research, vector-competence experiments, and high-resolution surveillance because it also prevents the target dilution associated with pooled specimens [39].

Conventional molecular assays or integrated nucleic acid, antigen, and CRISPR-based biosensors may be used to test individual mosquitoes. Such testing is costly and labor-intensive in large surveillance programs, so its use is most justified when a biosensor provides a clear benefit in speed, automation, cost, or epidemiological resolution [57].

#### 3.2.2. Mosquito Pools

Pooled testing can reduce the per-sample cost of high-throughput surveillance. RT-qPCR is still considered the gold standard technique, and other amplification-assisted and CRISPR-based biosensors are still being developed for use in the field [58]. Pool size involves a tradeoff: larger pools are more efficient but dilute the target, whereas smaller pools may be more sensitive but increase workload and cost.

The size of the pool is therefore an important design factor. Larger pools minimize the number of assays needed, but if the infectivity of a mosquito is low, dilution may result in higher Ct and in lower probability of detection when the pool size is too large. Generally, the smaller the pool, the better the probability of detection will be, although more assays will be needed. The optimum size of the pool depends on the anticipated infection prevalence, the species of mosquito, the sensitivity of the laboratory test, and the surveillance goal [59].

The pooled results should be analysed using appropriate epidemiological parameters (e.g., minimum infection rate, maximum likelihood estimation) and not as simple individual infection rates. Reports should document pool size, mosquito species, collection location and date, and storage conditions, as well as the testing method [60].

#### 3.2.3. Mosquito Homogenates

For most vector-based specimens, homogenization is mandatory prior to nucleic acid extraction or direct amplification. RNA yield and possible carryover of inhibitors may depend strongly on the homogenization method, buffer composition, bead-beating conditions, and clarification procedure. The mosquito tissues may also contain inhibitors of amplification or disruptors of antigen detection and biosensor readout [58].

Biosensors should therefore be tested in the context of the whole workflow, from homogenization to clarification, extraction, and target recognition to readout. Validation with purified targets alone is not sufficient since real infected and uninfected mosquito homogenates are needed [61].

#### 3.2.4. Field-Collected Mosquitoes and Species Identification

The variability in the age, feeding history, environmental exposure, microbial flora, and degree of degradation of field-collected mosquitoes may affect the assay’s performance compared to that of laboratory-collected mosquitoes [62]. Viral target is also difficult to interpret in engorged mosquitoes as a positive finding does not confirm a mosquito infection.

Correct identification of species is essential due to the vector competence of different species of Aedes and Culex [63]. Species-specific assays or molecular barcoding can be used in addition to morphological identification [64]. Future multiplex biosensors could detect viruses, identify mosquito species, determine collection location, and transmit results electronically; however, this would require different probes, multiple signal channels, and adequate interference control.

### 3.3. Animal and Environmental Specimens

Animal and environmental specimens are relevant when MBV surveillance is considered from a One Health perspective. However, they should be discussed selectively to avoid shifting the focus away from virological detection methods in human and vector samples [65].

#### 3.3.1. Sentinel Animals

Sentinel animals can provide early evidence of virus circulation. Birds are central to West Nile virus ecology, and avian surveillance can help detect enzootic transmission before human cases occur. Pigs are important amplifying hosts for Japanese encephalitis virus and may be used in surveillance systems in endemic regions. Non-human primates are relevant to selected sylvatic transmission cycles, such as yellow fever virus, but routine testing depends on regional ecology and public health infrastructure [66].

Their serum, blood, tissues, or swabs may be tested using conventional methods or nucleic acid, antigen, and antibody biosensors. However, viral kinetics, immune responses, and specimen composition differ across host species. Biosensors must therefore undergo host- and matrix-specific validation rather than relying on human-specimen data [67].

#### 3.3.2. Environmental Samples

Wastewater and water-associated surveillance remain less established for MBVs. Viral targets are often diluted or degraded, while organic material, salts, solids, and microbial products may interfere with extraction and biosensor signals. Concentration and purification are usually required, making the pre-analytical workflow more challenging than sensing itself.

Environmental biosensors should be evaluated as complete sampling-to-result systems. Detection of viral RNA does not confirm infectious virus or active mosquito-borne transmission. Environmental testing should therefore remain a complementary surveillance strategy until standardized methods and epidemiological thresholds are established [68].

## 4. Biosensing Platforms for Mosquito-Borne Virus Detection

In this review, an MBV biosensor is defined as an analytical system that integrates a biological recognition element, a physicochemical transducer, and a measurable readout, and that additionally satisfies three MBV-specific criteria distinguishing it from a generic laboratory biosensor. First, the recognition element must interact directly with an MBV-associated target or its amplification product. Second, signal transduction must be intrinsically coupled to the recognition event rather than requiring a decoupled downstream instrument for signal generation. Third, the platform must be designed for near-patient or field use, with a workflow and form factor compatible with point-of-care or field-deployable operation rather than dependence on centralized laboratory infrastructure. Amplification, microfluidics, and smartphone imaging are regarded as enabling technologies rather than biosensor components in their own right and are considered part of an MBV biosensor only when integrated with biorecognition and an intrinsically coupled signal-transduction step. This refined MBV-specific definition distinguishes functional biosensing platforms from conventional diagnostic assays and from generic laboratory biosensors, and it is applied consistently throughout this section.

### 4.1. Immunological and Lateral-Flow Biosensors

Immunological biosensors detect viral antigens or host antibodies through antigen–antibody recognition coupled to a visual, optical, or electrochemical output [69]. Lateral-flow biosensors are widely used at the point of care because they are rapid, inexpensive, portable, and require minimal equipment [70].

**Figure 2:**
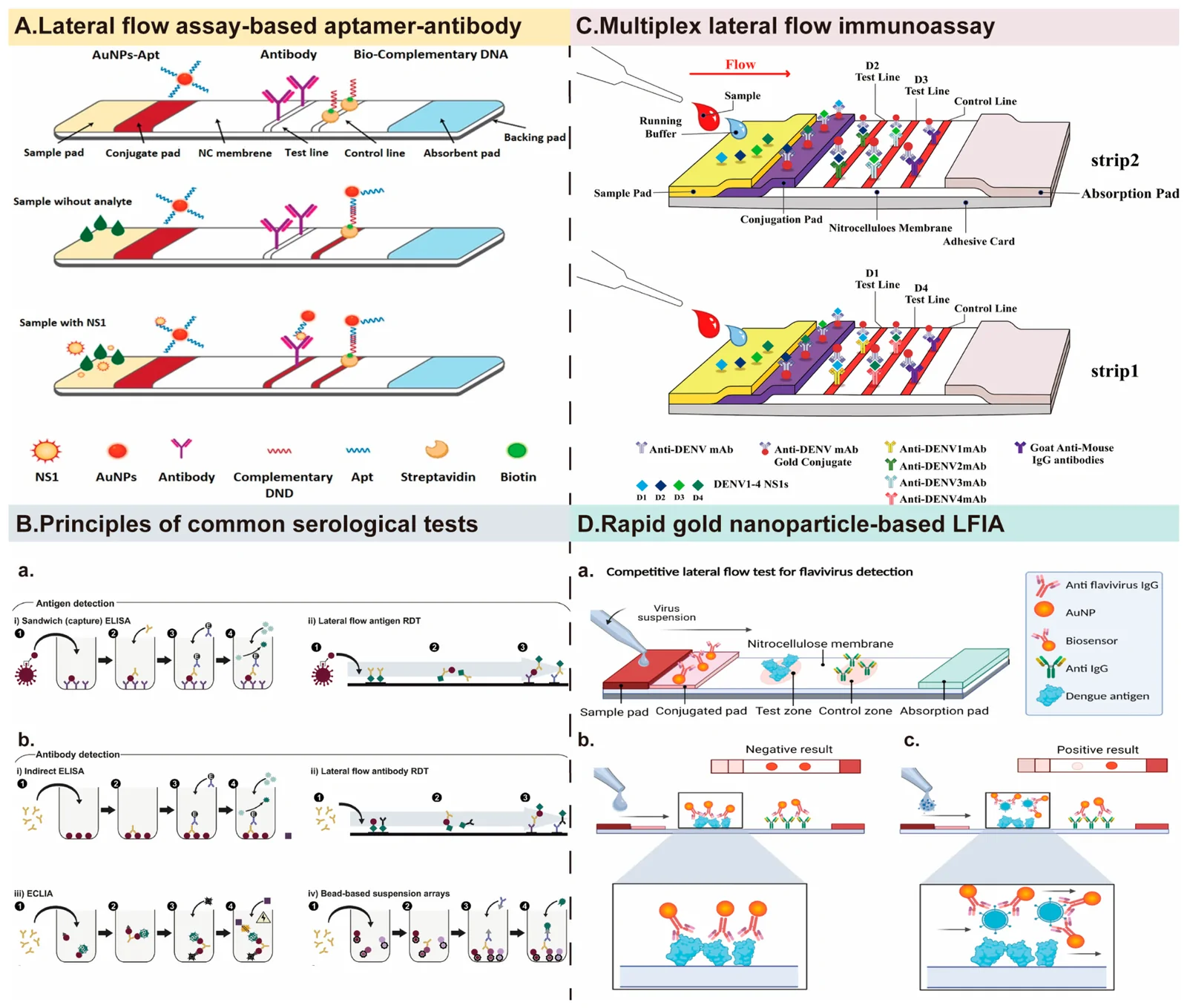
Schematic overview of the immunoassay-based detection technologies. (**A**) Schematic diagram of NS1 detection using a lateral-flow strip assay-based aptamer-antibody sandwich. Reprinted with permission from Ref. [87]. (**B**) Principles of representative antigen- and antibody-detection immunoassays. (**a**) Antigen detection: (**i**) sandwich enzyme-linked immunosorbent assay (ELISA), in which viral antigens are captured by immobilized antibodies and detected using labeled antibodies; and (**ii**) lateral-flow antigen rapid diagnostic test (RDT), in which viral antigens bind to labeled detection antibodies and are captured at the test line. (**b**) Antibody detection: (**i**) indirect ELISA, in which virus-specific human antibodies bind to immobilized viral antigens; (**ii**) lateral-flow antibody RDT, in which human antibodies bind to labeled viral antigens and are captured at the test line; (**iii**) electrochemiluminescence immunoassay (ECLIA), in which human antibodies form complexes with biotinylated and ruthenylated antigens; and (**iv**) bead-based suspension array, in which human antibodies bind to viral antigens coupled to magnetic beads. Reprinted with permission from Ref [88]. (**C**) Schematic illustration showing Dengue serotype NS1 multiplex LFIA [73]. (**D**) Scheme of the competitive lateral flow assay designed in this study for the detection of flaviviruses. (**a**) On the strip, the sample flows by capillarity to the different components, reacting with the biosensor on the conjugate pad producing a signal on the detection membrane. (**b**) If the sample does not contain flaviviruses, the biosensor flows to the detection membrane, binding to the analyte present in the test zone (DENV) and the secondary antibody against IgG present in the control zone, generating two signals (negative test). (**c**) If the sample contains flaviviruses, the biosensor–analyte complex is formed, which flows to the detection membrane, generating signals only in the control zone (positive test). Reprinted with permission from Ref [89].

Viral antigen tests detect viral components directly. Dengue NS1 antigen testing is the best-known example and can be used during early infection (Figure 2A) [71]. Its performance is not uniform. Commercial NS1 ELISA and lateral-flow assays have reported sensitivities of 82% and 72%, respectively, and positive results are associated with higher hemoglobin concentrations [72]. Most commercial antigen biosensors also cannot reliably determine DENV serotype [73].

Serological assays and biosensors detect IgM, IgG, or neutralizing antibodies (Figure 2B) [74]. Common formats include ELISA and immunochromatographic assays [75]. Lateral-flow immunosensors are a mature point-of-care format [76]; it requires no instrument and provides visual results. It is widely used for on-site screening of dengue NS1 antigen and dengue IgM/IgG antibodies [77]. Antibody testing has a timing limitation. IgM is often detectable only about 3–5 days after symptom onset, so early infection may produce false-negative results [78]. Conventional ELISA also requires laboratory equipment and longer workflows, although paper-based or microfluidic ELISA can reduce assay time and reagent consumption when integrated into biosensing devices [79].

Cross-reactivity remains a major limitation of flavivirus immunosensors. DENV, ZIKV, JEV, and other flaviviruses share conserved epitopes, which may produce false-positive antibody signals, especially in populations previously exposed through infection or vaccination. The use of virus-specific epitopes and engineered antigens could improve selectivity. Even so, these approaches require broader clinical validation.

Recent advances in biosensor design include multiplex lateral-flow strips, signal-enhancing nanomaterials, and portable readers (Figure 2C) [80]. Multiplex lateral-flow devices can test for dengue, Zika, chikungunya, and other causes of febrile illness in a single assay [81]. Concurrently, the incorporation of advanced labels, such as quantum dots [73], fluorescent microspheres [82], and engineered gold nanoclusters (Figure 2D) [83], has enhanced signal output and markedly improved analytical sensitivity. Portable readers allow semi-quantitative or quantitative interpretation [84]. Paper-based and microfluidic immunosensors may offer greater sensitivity than conventional colloidal-gold strips [85,86]. Together, these developments support a field-deployable model in which the sample is introduced, the signal is read visually or instrumentally, and a result is obtained within minutes.

Biosensor performance remains matrix-dependent. Hemoglobin and cells in whole blood may interfere with colorimetric signals, while mosquito homogenates can affect antibody binding and membrane flow. Saliva and urine offer non-invasive sampling but may contain lower target concentrations. Immunological biosensors should therefore be validated using representative specimens and samples from patients with related flavivirus infections.

### 4.2. Nucleic Acid Amplification-Assisted Biosensors

#### 4.2.1. Isothermal Amplification Methods

Nucleic acid testing improves early MBV detection by targeting viral RNA during the acute phase [90]. RT-PCR and RT-qPCR remain important reference methods but require thermal cycling and laboratory infrastructure [91,92]. They should not be classified as biosensors unless incorporated into a device that couples target recognition to signal transduction and readout.

Isothermal amplification methods, particularly LAMP and RPA, are more compatible with portable biosensing workflows [93]. However, it is important to note that LAMP and RPA reactions are themselves amplification-based chemical methods, rather than biosensors, because their initial products typically do not directly generate clear and interpretable signals. Therefore, these methods should be regarded as enabling or enrichment techniques used to increase the copy number of the target prior to detection. Only when their amplification products are coupled to a dedicated signal transduction interface such as a fluorescent probe, colorimetric indicator, electrochemical electrode, or lateral flow test strip, and convert target recognition into a measurable reading do they become components of amplification-assisted biosensors [94].

**Figure 3:**
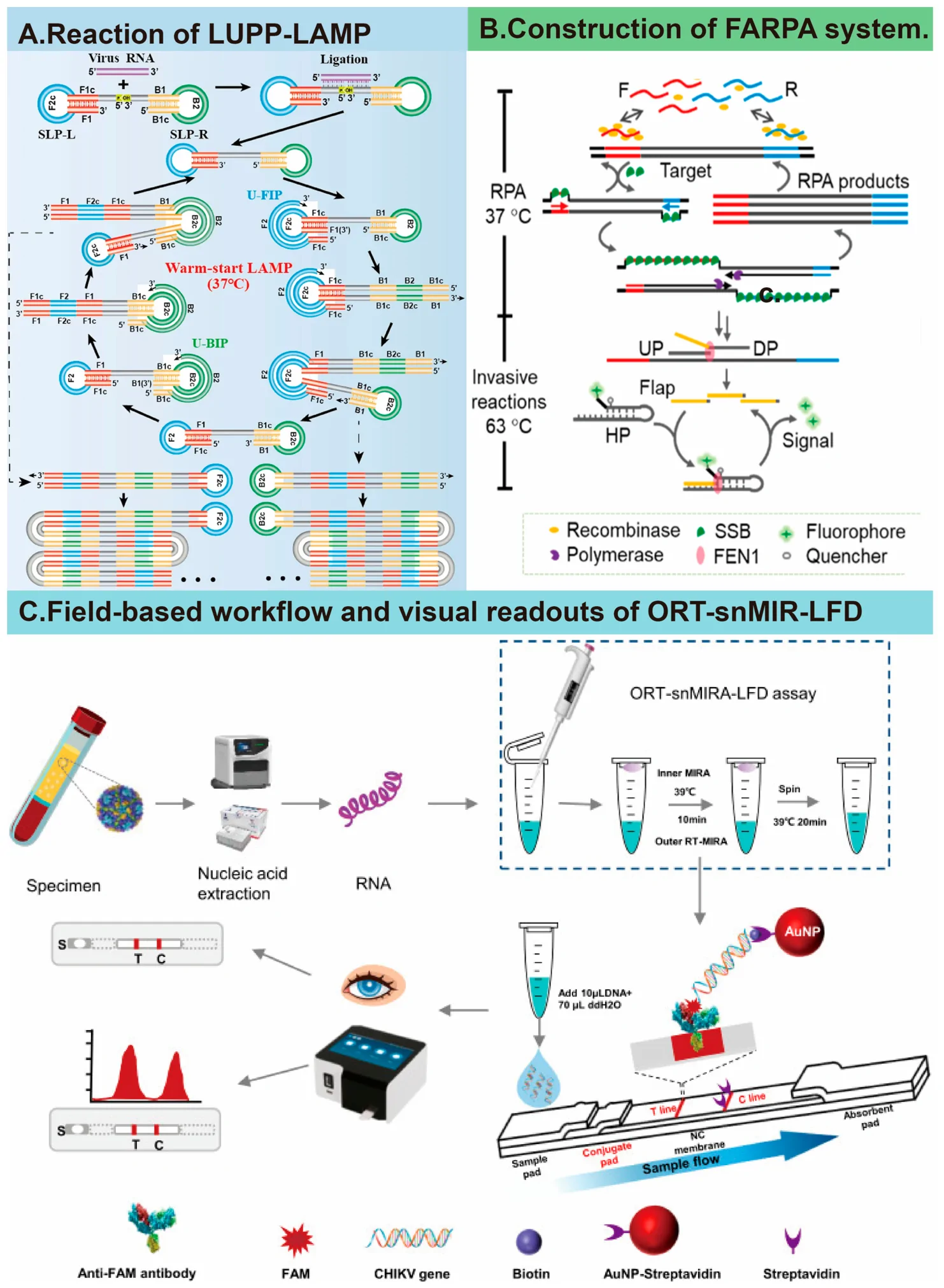
Schematic illustration of the isothermal amplification methods. (**A**) Overview of the LUPP-LAMP reaction. Reprinted with permission from Ref [100]. (**B**) Schematic of FARPA in which RPA and invasive reactions occurred sequentially and automatically by controlling the temperature needed for each reaction (37 °C for RPA, 63 °C for invasive reactions). Reprinted with permission from Ref [101]. (**C**) Detection and identification of CHIKV by one-tube, reverse transcription, semi-nested multi-enzyme isothermal rapid amplification, and lateral flow dipstick (ORT-snMIRA-LFD) assay. Schematic diagram of ORT-snMIR-LFD workflow. Reprinted with permission from Ref [102].

An integrated RT-LAMP lateral-flow biosensor developed for CHIKV achieved a detection limit of 598.46 copies/mL (Figure 3A) [95]. Field studies have also reported performance comparable to that of RT-qPCR in selected patient and mosquito specimens [96]. RPA typically operates at 37–42 °C and can yield results within 20–30 min, reducing both heating requirements and power consumption (Figure 3B) [97].

These advantages do not eliminate several persistent limitations. Isothermal biosensing remains susceptible to nonspecific amplification, complex primer design, carryover contamination, subjective visual interpretation, and restricted multiplexing capacity [98]. Inhibitors in whole blood and mosquito homogenates may further reduce amplification efficiency and weaken biosensor output. Performance established with purified RNA or serum should therefore not be assumed to apply directly to crude field specimens [99].

RPA may tolerate minimally processed specimens better than some LAMP workflows (Figure 3C), although its practical portability still depends on reagent stability, lyophilized formulations, and appropriate packaging. Field utility ultimately requires more than portable amplification. Sample preparation, amplification, signal generation, and objective readout must operate as a coherent biosensor-integrated workflow.

#### 4.2.2. CRISPR-Based Biosensors

Although isothermal amplification improves analytical sensitivity, nonspecific amplification may reduce result reliability [93]. CRISPR-based biosensors introduce an additional sequence-specific recognition step. After target recognition, Cas12 or Cas13 activates collateral cleavage of labeled reporters, producing fluorescent, colorimetric, electrochemical, or lateral-flow signals [103,104]. When coupled with RT-LAMP or RT-RPA, these systems follow an “amplify–recognize–report” workflow that combines high sensitivity with sequence-level confirmation [105].

**Figure 4:**
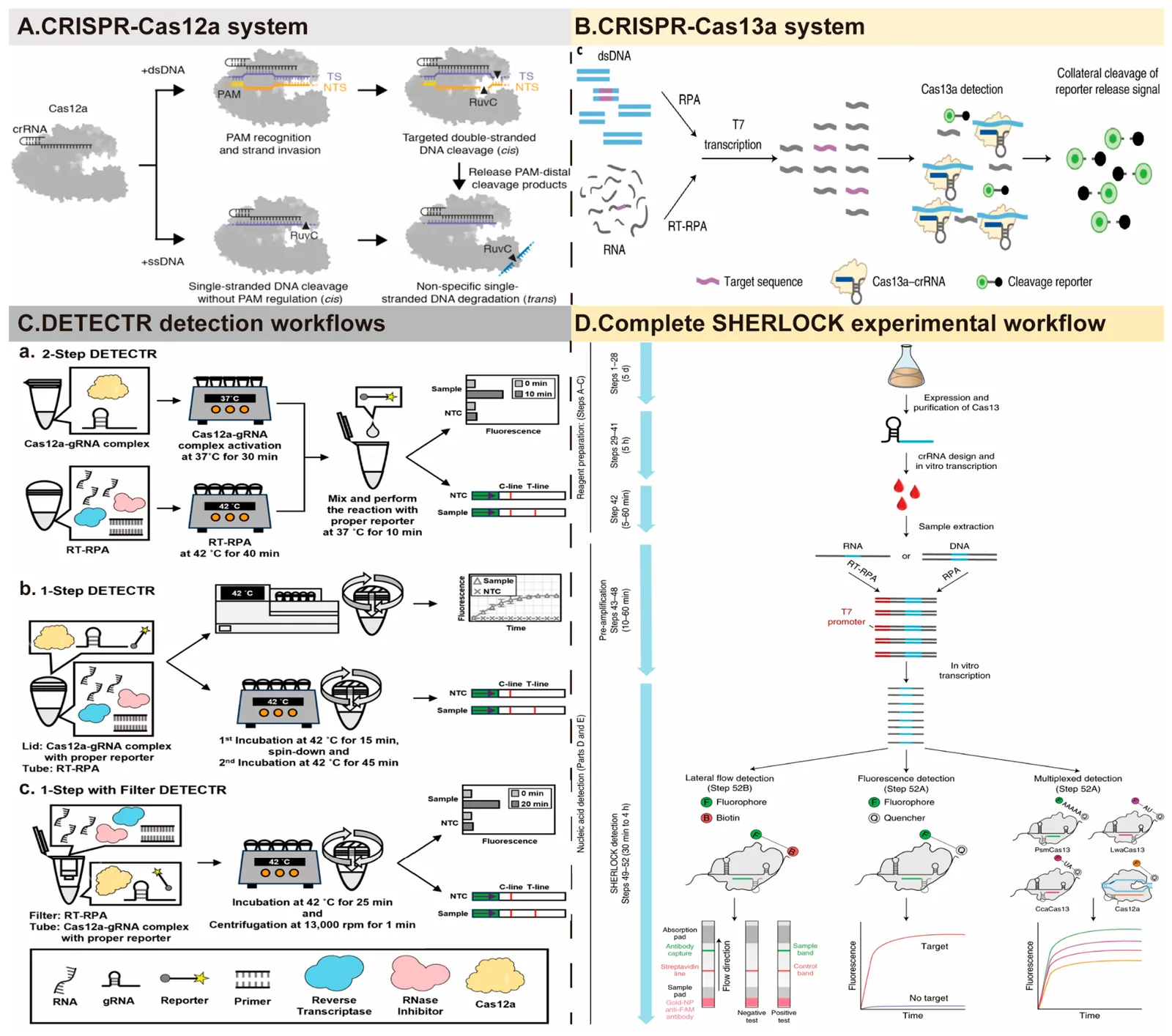
Schematic representation of integrating CRISPR-Cas within isothermal amplification for field-deployable detection. (**A**) Model for PAM-dependent and PAM-independent activation of cis and trans-cleavage by Cas12a. Reprinted with permission from Ref [106]. (**B**) (**c**) SHERLOCK detection assay. Schematic of SHERLOCK assay steps, starting with pre-amplification of either a DNA or RNA target input. Amplified targets are converted to RNA via T7 transcription and are then detected by Cas13–crRNA complexes, which activate and cleave fluorescent RNA reporters. Reprinted with permission from Ref [112]. (**C**) Schematic diagrams of 2-Step, 1-Step, and 1-Step with Filter DETECTR workflows. (**a**) 2-Step DETECTR; (**b**) 1-Step DETECTR; (**c**) 1-Step with Filter DETECTR. Reprinted with permission from Ref [113]. (**D**) Complete SHERLOCK experimental workflow. First, LwaCas13a is recombinantly expressed and purified in Escherichia coli (Steps 1–28). After crRNA design and in vitro transcription (Steps 29–41), sample extraction is performed to yield the target nucleic acid (Step 42). This sample is then used for RPA-based pre-amplification (Steps 43–48) and detection by Cas13 (Steps 49–52). Detection can be performed as fluorescence-based single or multiplex SHERLOCK reactions (Step 52A) or a single-plex colorimetric lateral flow reaction (Step 52B). Multiple targets can be detected within the same reaction using CRISPR–Cas13 enzymes with orthogonal cleavage preferences or by combining Cas13 with Cas12 in the same assay. Reprinted with permission from Ref [112].

DETECTR similarly uses Cas12a collateral cleavage for rapid nucleic acid biosensing (Figure 4A,C) [106]. SHERLOCK uses Cas13-mediated recognition and has demonstrated attomolar detection of DENV and ZIKV with single-nucleotide discrimination (Figure 4B,D) [107]. The HUDSON–SHERLOCK platform enabled testing of minimally processed body fluids within 2 h and differentiated DENV serotypes and ZIKV strains [108]. More recently, a one-pot RT-RPA/Cas12a biosensor detected DENV within 40 min, with a reported limit of 91.7 copies per test and no observed cross-reactivity with the evaluated non-target viruses [109].

This sequence-verification capability is valuable for differentiating DENV serotypes and closely related flaviviruses [110,111]. However, pre-amplification can increase contamination risk, while one-pot reactions require careful optimization of enzyme compatibility and reaction conditions. Biosensor performance may also be affected by inhibitors in whole blood and mosquito homogenates. Clinical and field validation should therefore assess the complete biosensor-integrated workflow, including sample preparation, amplification, CRISPR recognition, signal transduction, and readout. 

### 4.3. Integrated and Portable Biosensing Platforms

The integration of biosensors with microfluidics, paper-based devices, portable readers, and digital interfaces is advancing MBV detection from separate laboratory steps toward compact sample-to-answer systems [114]. Such biosensing platforms aim to reduce manual handling, contamination, turnaround time, and operator dependence.

#### 4.3.1. Engineering Integration and Biosensors

Microfluidic biosensors may include passive transport, mixing of samples and reagents, amplification, biorecognition, and signal detection, all in a single device [17,115,116]. Multiplex chips and lab-on-paper platforms are developed to detect DENV, ZIKV and CHIKV in conjunction with each other [117], and optical sensors and smartphone interfaces can extend capabilities by providing portable quantitative readout and data transmission [118,119].

**Figure 5:**
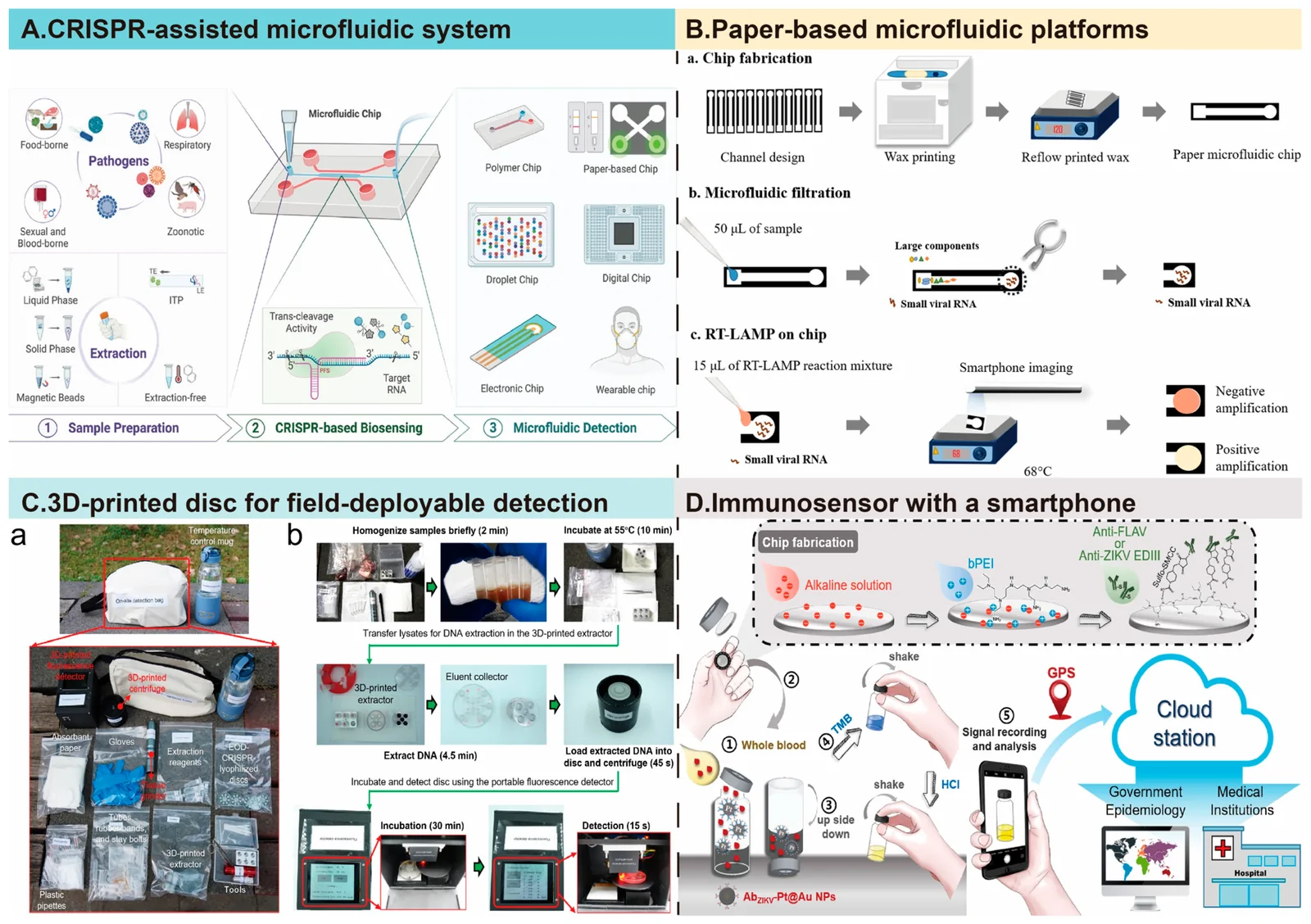
Integrated smart field-deployable platforms for virus detection and digital Surveillance. (**A**) Overview of CRISPR-based microfluidic system in infectious diseases diagnosis. Reprinted with permission from Ref [140]. (**B**) Schematic of paper microfluidic RT-LAMP assay. (**a**) The paper microfluidic chips were designed with SolidWorks (2015) and wax-printed on cellulose grade 4 paper. Printed wax patterns were melted and vertically flown through the depth of paper by placing the chips on a hot plate. The dark wax area represents the hydrophobic barrier and the bright area the hydrophilic area where liquid flows. (**b**) ZIKV-spiked samples of deionized water, tap water (undiluted), human urine (undiluted), or human blood plasma (diluted to 10% with Plasmolytic) were loaded onto the loading area of a paper microfluidic chip and spontaneously flowed through the channel via capillary action. Contaminants in the sample matrices were filtered during this process, while target RNA flowed at the same speed of bulk liquid. (**c**) The circular end (detection area) of the paper was cut, and the RT-LAMP reaction mixture was added onto it. This cut-out paper was placed on a hot plate at 68 °C for up to 40 minutes to achieve amplification. The color change of the paper is monitored in situ through capturing images with a smartphone. Reprinted with permission from Ref [141]. (**C**) Onsite bacteria detection platform and its performance. (**a**) Photos of the onsite detection bag and the list of materials. (**b**) Workflow of the detection platform. Reprinted with permission from Ref [142]. (**D**) Illustration of the fabrication processes and detection procedures as follows: (**1**) addition of spiked whole blood sample into VISZIKV; (**2**) securing of chip-containing lid; (**3**) shaking for 30 s, incubating upside-down for 15 min, and then transferring the lid to a new vial containing wash buffer (TBST); (**4**) transferring the lid to a new vial containing H2O2/TMB, securing the lid, shaking for 1 min, and allowing to stand for 3 min; and (**5**) signal recording by a smartphone for result determination, data storage, digital data transmission, and real-time geographical tagging of the results. Reprinted with permission from Ref [143].

However, a complete sample-to-answer system is still challenging to realize, since nucleic acid extraction is often carried out off-chip with variable target concentrations in crude samples and amplification/detection inhibitors. On-chip lysis and solid phase extraction have been shown, but recovery efficiency from whole blood or mosquito homogenates may be variable. Increased integration may also raise costs through greater reliance on external pumps or readers and more complex manufacturing (Figure 5C).

The inexpensive multistep assay can be conducted using paper with capillary flow [120]. These platforms can combine LAMP- or CRISPR-based recognition to a colorimetric, fluorescent or lateral-flow output (Figure 5A) [121,122]. One such approach is the use of an RT-LAMP paper-based biosensing system coupled with smartphone color analysis for CHIKV detection (Figure 5B) [123]. Their use is usually easy and inexpensive, but the flow control and quantitative accuracy are not as good as those of microfluidic chips.

The binding of antigen and antibody or the hybridization of nucleic acids to electrochemical biosensors produces measurable electrical signals, an ideal application for miniaturized and low-power devices [124]. Nanomaterials can help to enhance the surface area of the electrodes and boost the forces of the signals [125]. Commonly used designs are electrochemical immunosensors, nucleic acid biosensors, and disposable, screen-printed electrodes connected to portable readers [126,127,128].

For optical biosensors, fluorescence, colorimetry, plasmonic sensing, or surface-enhanced spectroscopy are used [129]. Fluorescent lateral-flow systems and imaging using a cell phone could enhance sensitivity without the subjectivity of visual interpretation [119]. The surface plasmon resonance is a technology for real-time, label-free biosensing in general; however, more complex and costly instruments are required [130].

#### 4.3.2. Intelligent Readout and Data Connectivity

MBV biosensing is slowly moving towards more connected digital readout [131]. Smartphones are capable of measuring colorimetric or fluorescent signals, automating classification of results, and reporting time- and location-specific data to surveillance networks (Figure 5D) [119,132,133,134]. These functions can be coupled with multiplex biosensors to aid concurrent testing of DENV, ZIKV, and CHIKV and to enhance epidemiological reporting [135].

Less harmful methods of vector surveillance are possible by using mosquito saliva or excreta and nucleic acid-stabilization cards [136,137]. These approaches are biosensor-based only if target recognition is coupled with a signal transduction and measurable output. Similarly, FTIR, NIR, and Raman spectroscopy are analytical sensing technologies without the addition of biological recognition and therefore are not biosensors. The external validation of the algorithmic classification by these techniques is necessary across locations, mosquito species, and specimen matrices [138].

Machine learning and deep learning tools may improve strip interpretation and reduce operator variability [139]. Nevertheless, connected biosensors must also address calibration, data security, device compatibility, and algorithm bias. Ultimately, field value depends not only on analytical sensitivity but also on the ability of a biosensor-integrated platform to process crude samples reproducibly while remaining rapid, affordable, stable, and easy to operate. A comparative summary of the point-of-care detection methods discussed is given in Table 3.

**Table 3:** Comparative performance of point-of-care and reference detection methods for MBVs.

Method	Target	Temperature	Sample Type	Assay Time	LOD/Sensitivity	Advantage	Limitation	Ref.
ELISA	DENV	Room temperature	Serum, plasma	5–7 h	82%	High detection throughput enables suitability for batch screening	The operation is complex and time-consuming, making it unsuitable for on-site applications	[72]
LFIA	DENV 1–4	Room temperature	Serum, mosquito homogenate	15–30 min	90.0% (DENV1) 88.2% (DENV2) 82.6% (DENV3) 83.3% (DENV4)	It is the most commercially mature, requires no equipment, and produces results within 15–30 min	Residual cross-reactivity with other flaviviruses, lower sensitivity than molecular detection	[73]
ELISA	ZIKV, DENV1, WNV	37 °C	Serum, plasma	~7 h	100%	Mutant VLP and rOD ratio substantially reduce cross-reactivity	Requires multiple antigens; relatively small sample sizes per infection panel	[144]
RT-qPCR	ZIKV	Thermal cycling	Serum, urine, plasma	1–2 h	10–25 copies/rxn	Established gold-standard method; high sensitivity and specificity	Requires thermal cycler and trained personnel	[145]
RT-LAMP	CHIKV	63 °C	Serum	60 min	20 copies/rxn	Rapid, cost-effective, visual readout; instrument-free	Complex primer design; risk of aerosol contamination	[146]
RT-RPA-LFD	DENV 1–4	39 °C	Serum, saliva, urine	35 min	10–1000 copies/mL	Non-invasive sampling; rapid	Lower sensitivity than PCR; cannot differentiate serotypes	[147]
SHERLOCK v2	ZIKV, DENV	37 °C	Blood, serum, urine, saliva	15–60 min	2 aM/µL	Highly sensitive and specific; multiplexable (up to 4 targets); quantitative; portable	Multi-step workflow (RPA pre-amplification + Cas13 detection); crRNA design is critical	[112]
HUDSON/RT-RAA/CRISPR-Cas12a	DENV2	39 °C	Serum, mosquito homogenate	~1 h	100 copies/µL	Extraction-free; rapid; point-of-care	Validated for DENV-2 only; requires further evaluation across serotypes and clinical matrices	[148]
RT-RPA/CRISPR-Cas12a (One-pot)	DENV 1–4	39 °C	Whole blood	50 min	10 ng/rxn	One-pot reaction reduces contamination risk; visual readout; pan-DENV detection	Slightly reduced sensitivity compared to two-step methods; requires careful reagent optimization	[149]
Integrated Microfluidic System	DENV2	Thermal cycling	Serum	30 min	21 pg/mL	Fully integrated sample-to-answer on a single chip; minimizes contamination	Requires specialized chip fabrication and instrumentation	[150]
Polymer Microchip	ZIKV, DENV, WNV	65 °C	Serum, mosquito homogenate	~3.5 h	N/A	Low-cost disposable platform; suitable for field deployment	Still requires PCR amplification; chip fabrication adds complexity	[151]
Paper Microfluidic Chip & Smartphone	ZIKV	68 °C	Plasma	15 min	1 copies/µL	Rapid; smartphone-based fluorescence detection; integrated paper chip; low cost	Requires external heating element; image processing algorithm needed	[141]
Fiber-Optic SPR Platform	DENV	Room temperature	Serum	20–25 min	0.06 μg/mL	Portable; reusable sensor; real-time label-free detection	Requires fiber-optic setup; not yet commercialized	[152]
Enzyme-Free Vial Immunosensor & Smartphone	ZIKV	Room temperature	Urine, serum, whole blood	20 min	1pg/mL	Enzyme-free; low cost; smartphone readout; suitable for field surveillance	Requires antibody conjugation; dependent on optical setup quality	[88]
SPCE Electrochemical Immunosensor & Smartphone	JEV	Room temperature	Serum, urine	30 s	0.45 fM	Portable; ultrasensitive; smartphone interface; validated in clinical samples	Requires electrode surface modification; limited sample validation	[153]
Paper-Based Nucleic Acid Testing System	ZIKV, DENV, CHIKV	65 °C	Serum	~1 h	1–10 copies/rxn	Simple, low-cost paper-based LAMP platform; minimal equipment required	Separate amplification and detection steps; not fully integrated	[154]

Notes: The performance values summarized in this table were obtained from different studies using different units (e.g., copies/mL, PFU, ng, or %), specimen types, and experimental conditions, and some values represent analytical sensitivity while others represent clinical sensitivity. Because these differences preclude direct standardization, the values listed should not be interpreted as head-to-head comparisons across assays or platforms.

### 4.4. Sample Processing as a Determinant of Biosensor Performance

Advances in isothermal amplification, CRISPR-based recognition, electrochemical sensing, and microfluidics can improve analytical performance and portability. However, these advantages may be offset by inefficient target release and incomplete removal of matrix-derived inhibitors. These limitations can result in higher detection limits, greater variability, and increased false-negative rates. The performance of an integrated biosensor may therefore be overestimated when it is evaluated using purified RNA or targets spiked into buffer [15,155].

Matrix effects vary substantially among specimen types. Proteins, lipids, pigments, and tissue debris in mosquito homogenates can interfere with nucleic acid amplification, membrane flow, and optical or electrochemical measurements. Hemoglobin, heparin, immunoglobulins, and cells in whole blood can act as background signals or polymerase inhibitors and should therefore be minimized or controlled for. Saliva and urine can be collected noninvasively, but both matrices may contain enzymes, salts, urea, or pH-dependent factors that interfere with target recovery or detection [51]. Environmental water causes another set of issues as particles, organic matter and humic compounds can interfere with target recovery and the biosensing signal [43,44].

In addition to biochemical inhibition, crude samples present challenges related to physical flow, which are particularly detrimental to microfluidic and paper-based detection platforms. Variations in whole blood viscosity and hematocrit may slow or unevenly distribute capillary flow on paper-based substrates; meanwhile, cell debris and mosquito tissue particles may block microfluidic channels or contaminate filter membranes, leading to incomplete sample delivery and unstable signal responses [155]. These effects are independent of and additive to the inhibitory effects during enzymatic amplification; when validation of detection platforms is conducted using clarified samples or samples diluted with buffer, the aforementioned issues are often underreported. Mitigation strategies include using integrated plasma separation membranes to reduce the cell load; incorporating a pre-filtration zone to capture large particulate impurities before the detection zone; applying hydrophilic surface treatments to maintain stable capillary action; and optimizing channel geometry to minimize narrow sections prone to blockage [156].

Traditional silica column extraction typically yields fairly pure nucleic acids but requires repeated washing and elution steps, controlled reagents, and centrifugation or vacuum systems [156]. More readily adapted to automated cartridges is magnetic-bead extraction, which is more expensive and must be carefully controlled for buffer conditions and bead carryover [157]. For CRISPR- and amplification-assisted biosensing, heat- or chemical-based lysis could simplify the process, although it depends on the type of specimen, the viral load, the target stability, and resistance to enzymes [158]. Extraction-free detection minimizes handling and could lower the potential for contamination but may be insufficient for certain samples (high inhibitor content or low concentration of target) and specimens.

The field-deployable pretreatment module should minimize the need for pipetting, equipment, cold-chain management, and user handling [15]. Ideally, the module should stabilize the target at room temperature, complete the necessary processing steps, and produce material suitable for isothermal amplification, CRISPR-based biosensing, electrochemical detection, or paper-based readout [159]. Membrane filtration, magnetic capture, solid phase extraction in paper or microfluidic formats, and thermochemical lysis are potential approaches [155].

Another option is to pair minimal processing with inhibitor-tolerant amplification and CRISPR chemistry [159]. This strategy may sacrifice some analytical sensitivity, but the trade-off could be justified if it improves speed, usability, and contamination control [160]. Sample preparation should not be treated as an independent pre-analytical step. It is part of the biosensing workflow and must be optimized for the intended application, whether clinical diagnosis, mosquito surveillance, environmental monitoring, or outbreak screening. A comparative summary of these sample processing strategies is presented in Table 4.

## 5. Current Challenges and Limitations

MBV diagnostics are shifting from laboratory-centered assays toward portable biosensing systems. However, successful field deployment generally depends on factors beyond analytical sensitivity. Major limitations include sample-to-answer integration, multiplex performance, representative field validation, standardization, affordability, supply-chain resilience, and connectivity with public-health surveillance. These factors should be considered across the complete biosensor workflow, from specimen input and biorecognition to signal transduction, interpretation, and reporting.

### 5.1. Sample-to-Answer Integration

As discussed in Section 4.4, specimen-dependent target recovery, matrix inhibition, and fluid-transport behavior constrain field biosensing. Accordingly, sample-to-answer systems must be designed for the intended specimen and use case rather than around a universal pretreatment protocol [160,161]. 

The principal engineering challenge is to incorporate target release, inhibitor removal, biosensor-based detection, and waste containment into a simple closed system, while also accommodating the physical flow demands of the chosen platform for microfluidic and paper-based formats in particular; this includes maintaining consistent capillary or pressure-driven flow and preventing channel clogging or membrane fouling under crude specimen loading. A platform that requires off-chip extraction, repeated pipetting, or temperature-sensitive reagents has limited field readiness, even when its analytical sensitivity is high. Many reported biosensors remain proof-of-concept devices evaluated with purified targets or extensively processed specimens. Future biosensor-integrated platforms should therefore prioritize enclosed, low-step sample processing and demonstrate performance with minimally processed clinical and vector samples [159,162].

### 5.2. Multiplex Biosensing and Broad-Panel Capacity

Because several MBVs can co-circulate and produce overlapping clinical manifestations, multiplex detection may be particularly valuable in settings where differential diagnosis or broad vector surveillance is required. Panels should reflect local epidemiology and may include DENV, ZIKV, CHIKV, JEV, WNV, or YFV, as well as DENV serotypes when clinically or epidemiologically relevant.

Multiplex biosensing introduces competition among recognition probes or amplification reactions, nonspecific signals, channel cross-talk, and target-dependent shifts in detection thresholds [110]. These effects may be more pronounced in crude specimens, coinfections, and samples containing low-abundance targets. Spatially separated reaction zones, barcoded probes, microfluidic compartmentalization, and multichannel optical or electrochemical transducers can reduce signal overlap, but they increase device complexity and cost [163].

Each multiplex platform should therefore be evaluated using individual targets, mixed targets, coinfected samples, negatives, and specimens containing related viruses. Target combinations should be epidemiologically focused rather than maximized without clinical justification. At present, many biosensor-based assays remain limited to one pathogen or a small panel, and broader but epidemiologically adaptable platforms may offer additional value for differential diagnosis and vector surveillance [164].

### 5.3. Balancing Biosensor Performance and Field Utility

Few biosensors can optimize all these properties simultaneously; field-deployable platforms must therefore balance sensitivity, specificity, turnaround time, simplicity, throughput, and cost [165]. Intentional uses also differ in their priorities: clinical biosensing needs to provide timely and accurate assistance to patient management [166], while vector surveillance requires emphasis to be put on throughput, cost and the ability to withstand environmental fluctuations [167]. Home use is particularly important for simple collection, easy interpretation and protection from operator error [168]. Cost assessment should consider not only the sensing element but also sample-processing materials, reagents, disposable components, instrumentation, packaging, quality-control testing, storage, and distribution. Reagent stability and reduced dependence on cold-chain transportation are therefore important for both field utility and commercial feasibility.

Biosensor development should follow relevant quality frameworks, including ISO 15189 for medical laboratories, ISO 13485 for medical-device quality-management systems, and ISO 14971 for medical-device risk management. These standards support quality and risk management but do not themselves constitute regulatory authorization. Applicable CLSI guidance should inform the evaluation of detection capability, precision, interference, and qualitative test performance. Before pivotal studies begin, developers should define the intended use, target population, specimen type, operator requirements, biosensor cut-off, and criteria for invalid results. The applicable regulatory pathway depends on the intended use, device risk, and jurisdiction and may include relevant US Food and Drug Administration pathways, the European Union In Vitro Diagnostic Medical Devices Regulation, and applicable WHO mechanisms. Regulatory routes such as the WHO Emergency Use Listing may facilitate deployment during eligible public-health emergencies, but they do not remove the need for post-market surveillance, lot verification, and continuing quality control.

Many emerging platforms lack representative clinical or field evidence as mentioned in Section 4. Laboratory test results do not guarantee performance under different temperature, humidity, light, power supply and operator experience conditions. Thus, the role of translation should be evaluated, not just as a recognition chemistry or transducer sensitivity but over the entire sensor-integrated workflow. Standardized field tests are required to assess its reproducibility from operator to operator, site to site, specimen matrix to specimen matrix and manufacturing lot to manufacturing lot [169].

### 5.4. Cost, Accessibility, and Operational Sustainability

Many biosensors based on the CRISPR technology, microfluidic cartridges, electrochemical sensors, and innovative optical platforms can minimize hands-on time but can also require proprietary reagents, special fabrication, separate readers, and cold-chain storage [170]. Estimates of cost based on disposable reagents will therefore tend to underestimate the resources needed for routine use [154].

The total cost of biosensing exceeds the cost of the test as well because specimen collection, sample preparation, reader procurement and maintenance, quality control, staff training, repeat or confirmatory testing, data management, and waste disposal should be taken into account. Shelf life of reagents, serviceability in the region, ability to produce in large quantities, and supply chain strength in the face of high temperature and humidity also influence accessibility. A platform with slightly higher per-test cost can be justified when it reduces the time to treatment and/or eliminates repeat visits or facilitates multiplex surveillance. Instead of only considering the cost of the reagent, an economic assessment should consider the cost of a correct diagnosis or surveillance result and should incorporate the prevalence of the disease, the volume of testing, pathways for referral, and the repercussions of misdiagnosis.

### 5.5. Standardization of Evaluation and Result Interpretation

Meaningful comparison among MBV biosensors is hindered by differences in specimen type, sample processing, target material, reference method, disease stage, signal threshold, and statistical analysis. Detection limits derived from synthetic oligonucleotides, plasmids, cultured viruses, or buffer-spiked samples are not directly comparable, nor should they be interpreted as evidence of clinical sensitivity [171].

Biosensor development should follow the relevant quality frameworks, including ISO 15189 for medical laboratories, ISO 13485 for medical-device quality systems, and ISO 14971 for risk management. Applicable CLSI guidance should inform the evaluation of detection capability, precision, interference, and qualitative test performance. Before pivotal studies begin, developers should define the intended use, target population, specimen type, operator requirements, biosensor cut-off, and criteria for invalid results. Regulatory routes such as the WHO Emergency Use Listing may facilitate deployment during outbreaks, but they do not remove the need for post-market surveillance, lot verification, and continuing quality control.

Reader-assisted interpretation can reduce subjectivity in colorimetric and lateral-flow biosensors. Digital analysis, however, introduces further requirements, including calibration, software version control, cybersecurity, and consistent performance across smartphones, cameras, and lighting conditions. Algorithms and readers must therefore be validated as parts of the complete biosensing system, not as independent accessories [172].

### 5.6. Connected Biosensors and Digital Surveillance

When connected, biosensors can link test results to time, location, specimen type, device identity, and quality control data to facilitate rapid reporting, spatial mapping, and quicker outbreak response [131]. Smartphone-based readers are especially valuable since they can measure optical signals, can assist the user in navigating the testing protocol, and can report the results to a clinical or vector-surveillance network [173].

Standardization of the data format, interoperability with existing reporting systems and traceability between devices, reagent lots, calibration records and software versions are essential for good implementation. For systems with poor connectivity, it is important to enable encrypted offline storage and delayed sync. From the design phase forward, privacy protection, access control, cybersecurity, and responsible use of geolocation data are necessary.

Even with connectivity, biased sampling, incomplete testing coverage, or shifts in health-seeking patterns cannot be fully offset by connectivity alone. The analysis of connected biosensing platforms should therefore go beyond diagnostic effectiveness and include data completeness, reporting time, representativeness, interoperability, and the capability to trigger timely public-health action [174]. The utility of an MBV sensor ultimately lies in its ability to deliver reliable, meaningful, and securely interconnected results in the field.

## 6. Conclusions and Future Perspectives

**Figure 6:**
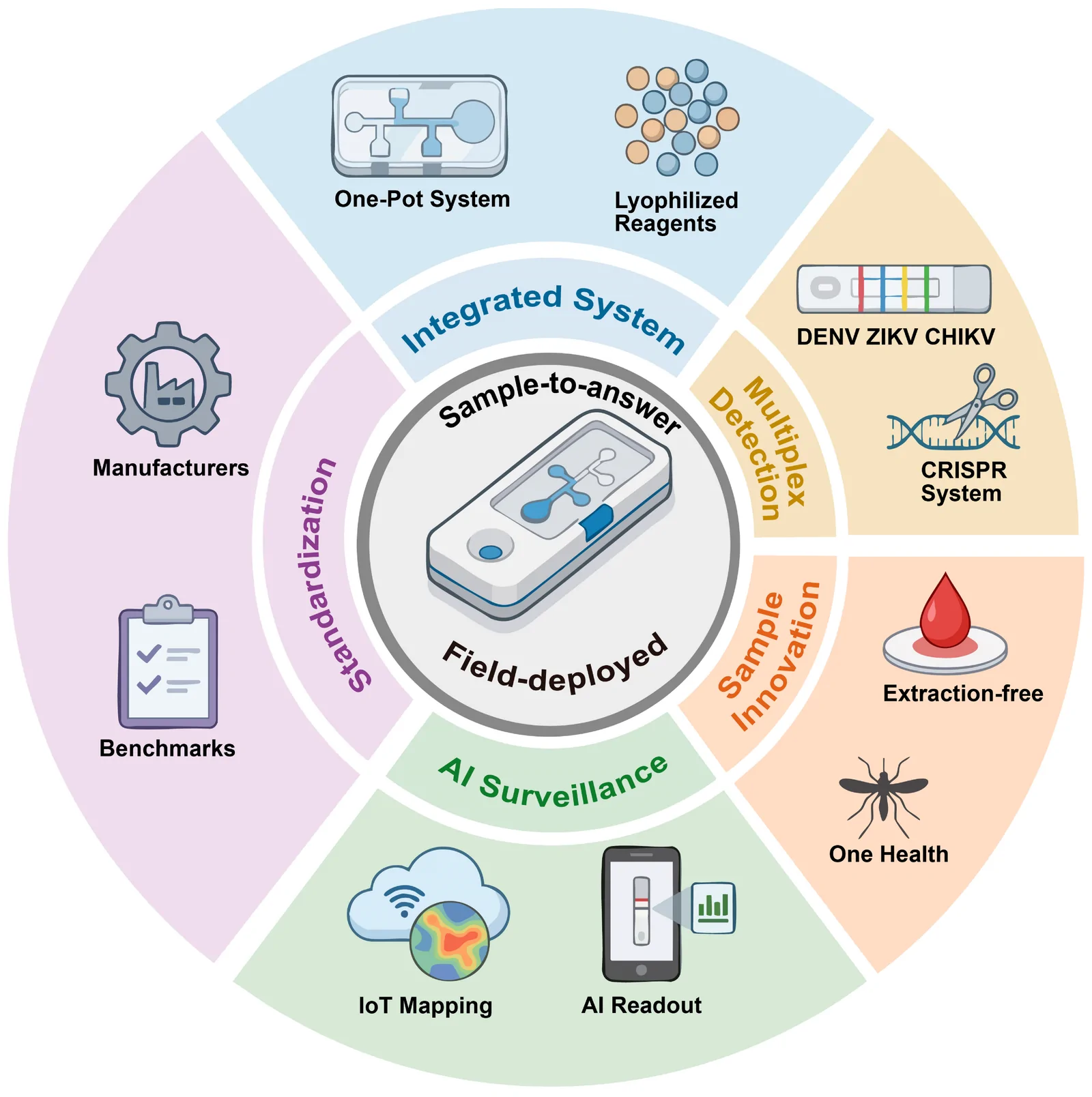
Future perspectives in field-deployable detection technologies for MBVs.

Future development of MBV biosensors should prioritize effective sample-to-answer integration, matrix tolerance, representative validation, affordability, data security, and, where appropriate, multiplex detection and digital connectivity (Figure 6). Platforms that combine reliable biorecognition technology, well-defined signal transduction mechanisms, automated or objective interpretation capabilities, and operational and maintenance capabilities suitable for field use into a comprehensive workflow often deliver the greatest practical value. Building on the limitations discussed above, we highlight several concrete directions that could accelerate this transition from prototype to field-deployable biosensor.

### 6.1. Integrated Sample-to-Answer Biosensing

Future sample-to-answer systems may integrate sample metering, reagent storage, reaction control, target detection, and result interpretation within an enclosed cartridge [100]. Their translation will require balancing analytical performance against cartridge complexity, manufacturing consistency, cost, power consumption, assay time, and ease of use. Highly integrated designs may reduce manual operation and contamination risk but may also increase manufacturing and quality-control requirements. Modular configurations may offer greater adaptability for clinical diagnosis and vector-surveillance applications.

This integration can help to cut hands-on time and minimize contamination risk and operator dependency. However, several critical engineering challenges still remain, such as stable fluid control, ambient-temperature reagent stability, waste containment, and compatibility with minimally processed specimens [175]. Reagent stability poses a unique technical barrier in tropical regions, where high temperatures and humidity, intermittent power supplies, and lengthy “last-mile” delivery routes often make the consistent maintenance of a cold chain impractical. Therefore, whenever feasible, enzymes, antibodies, CRISPR-associated reagents, and fluorescent reporters should be formulated as thermostable or lyophilized reagents and validated under the temperature and humidity conditions expected during storage, transportation, and on-site use.

Digital connectivity could further link field results with surveillance systems through time-stamped and, where appropriate, georeferenced reporting. Such systems should support standardized data formats, offline operation with later synchronization, secure transmission, and protection of patient or location-related information. Connectivity should complement rather than complicate the core diagnostic workflow, particularly in settings with limited network or power infrastructure [170].

### 6.2. Multiplex Biosensors and One Health Surveillance

Single-target tests may remain useful in specific clinical or surveillance settings; however, in regions where multiple MBVs circulate simultaneously and cause overlapping clinical manifestations, the diagnostic value of such testing is limited. Multiplex biosensors should therefore prioritize epidemiologically relevant target panels and, where justified by the intended clinical or surveillance use, may also incorporate serotype- or lineage-level discrimination. Spatially encoded lateral-flow devices, microsphere arrays, parallel CRISPR-effector systems, microfluidic partitioning, and multichannel electrochemical or optical sensors may help reduce target competition and signal overlap [176,177].

Surveillance need not be restricted to human specimens. A One Health analysis of human, mosquito, animal, and selected environmental samples may give a fuller picture of the circulation of the virus. Deployment of biosensors in clinics, vector traps, or surveillance laboratories could help with earlier recognition of outbreaks by providing georeferenced results [178]. While a shared platform may provide similar performance in common applications, different combinations of specimen type and target will require matrix-specific sample processing.

### 6.3. Matrix-Tolerant Sample Processing and Alternative Sampling

Inhibitors in blood, saliva, urine, mosquito homogenates, or environmental specimens may impact the recovery of targets or signal generation. Future integrated systems should therefore involve simple pretreatment techniques like membrane filtration, thermochemical lysis, magnetic capture, or microfluidic solid-phase extraction and reagents that do not require a cold-chain supply [148].

Extraction-free and inhibitor-tolerant chemistries may further simplify testing but can result in lower target recovery; therefore, their applicability should be validated for each intended use rather than assumed. Non-invasive samples like saliva and urine could facilitate decentralized testing, while mosquito excreta or saliva collected via sugar-baited traps could provide non-destructive vector surveillance [179]. The usefulness of these options will be determined by target kinetics, consistency of collection, and the compatibility of the downstream biosensors.

### 6.4. AI-Assisted Readout and Connected Biosensor Networks

The interpretation of the colorimetric, fluorescence, and lateral-flow biosensors can be made more objective by automated analysis. Machine learning algorithms and smartphone imaging have the potential to measure signals reliably under variable field conditions, consistent with the connectivity and ease-of-use metrics of REASSURED [15]. Stable AI-aided interpretation remains reliant on standardized image capture and appropriate calibration, representative training data, external validation, and measures against biases related to specific devices or groups.

Biosensor networks connected with smart mosquito traps, geospatial mapping, environmental monitoring, and public-health dashboards could allow near-real-time risk evaluation and outbreak response [180,181]. These networks are expected to offer interoperable data formats and offline functionality as well as traceability, cybersecurity, and privacy protection. AI-generated outputs should be used to assist quality control, epidemiological judgment, and confirmatory testing, rather than to replace them.

### 6.5. Standardization and Translation

Translation continues to be constrained by variation in specimens, reference methods, viral strains, detection thresholds, and statistical reporting. Common performance criteria are needed for analytical and diagnostic sensitivity, specificity, reproducibility, matrix tolerance, stability, usability, and invalid-result rates. Reference panels should contain clinically relevant target concentrations, inhibitor-rich matrices, related viruses, and representative diversity among serotypes or genotypes. For sequence-dependent detection platforms, including amplification-based methods and CRISPR-assisted biosensors, analytical inclusivity testing should be performed for all four DENV serotypes and representative circulating genotypes, as well as epidemiologically significant ZIKV and CHIKV lineages. Mutations within the primer-, probe-, or guide-RNA-binding regions may reduce amplification or recognition efficiency and lead to false-negative results due to sequence mismatches. Therefore, the coverage assessment should combine periodically updated computational sequence analysis with experimental validation using representative isolates, cultured samples, or well-characterized synthetic reference materials at clinically relevant concentrations.

Regulatory pathways, including the WHO Emergency Use Listing and relevant national frameworks, can accelerate outbreak access, but biosensor-based diagnostics still require manufacturing quality control, lot verification, and post-market surveillance [182]. Commercial translation also requires reproducible performance at the intended production scale. Variability in sensor fabrication, surface functionalization, biorecognition reagents, reagent deposition, and device assembly may lead to lot-to-lot differences in analytical performance. Therefore, critical materials and manufacturing processes should be controlled through an appropriate quality-management system, with acceptance criteria established for production lots.

Regulatory and manufacturing strategies should be considered early in development. Relevant considerations include ISO 13485 and ISO 14971, applicable FDA pathways, the EU IVDR, and WHO mechanisms where appropriate. In parallel, cost-of-goods analysis should account for reagents, disposable cartridges, instruments, packaging, quality-control testing, storage, and distribution rather than the sensing component alone. Scalable fabrication, reagent stability, and reliable sourcing of critical components are particularly important for maintaining affordable and sustainable production. Successful translation is also likely to depend on collaboration among academia, industry, public-health agencies, clinicians, and vector-control programs. Academic prototypes intended for translation should be designed with scalable fabrication, supply-chain resilience, and field implementation in mind [170].

Taken together, advancing the development of point-of-care MBV biosensors over the next few years requires a focus on the following four specific priorities. First, efforts should be made to promote the adoption of uniform standards for reporting analytical and clinical performance metrics in field applications to enable meaningful cross-study comparisons. Second, AI/ML-assisted reading technologies should be combined with digital connectivity to reduce the impact of operator-dependent interpretation and support real-time epidemiological reporting. Third, prospective, multicenter clinical validation should be conducted to replace reliance on synthetic targets or small convenient samples. Fourth, manufacturing scalability, quality systems, stability under anticipated tropical storage and distribution conditions, and analytical inclusivity across relevant viral serotypes, genotypes, and lineages should be incorporated from the prototyping stage rather than addressed as afterthoughts.

Overall, CRISPR-based biosensors, microfluidics, electrochemical sensing, automated readout, and connected reporting are expanding the capabilities of field-deployable MBV diagnostics. Their practical impact is likely to depend on whether they can provide accurate, affordable, and actionable sample-to-result performance under real-world conditions, rather than on analytical sensitivity alone. Early consideration of manufacturing reproducibility, quality systems, regulatory planning, and sustainable production costs will be essential for translating promising academic prototypes into clinically and operationally useful products.

## Figures and Tables

**Table 1:** Virological targets and diagnostic windows for MBV detection.

Target	Typical Detection Window	Suitable Methods	Main Applications	Main Limitations
Viral RNA	Early acute phase (DPO 0–5)	RT-qPCR, RT-PCR, RT-LAMP, RT-RPA, CRISPR-based assays, sequencing	Early diagnosis, confirmation of acute infection, viral load estimation, vector surveillance	Strongly dependent on sampling time, RNA preservation, extraction efficiency and inhibition control
Viral antigen	Acute phase (DPO 0–7)	ELISA, lateral-flow assay, immunosensor, chemiluminescent assay	Rapid acute dengue diagnosis, field screening	Sensitivity varies by virus, serotype, infection stage, immune status and assay format
IgM	Approximately (DPO 3–7)	MAC-ELISA, IgM ELISA, LFA, multiplex immunoassay	Diagnosis after RNA/antigen decline, outbreak investigation	Early false negatives; flavivirus cross-reactivity; persistence may complicate interpretation
IgG	Later phase (DPO 7–14)	IgG ELISA, immunofluorescence assay, neutralization assay	Seroprevalence, immune status, secondary infection assessment	Cannot reliably distinguish current from past infection using a single sample; cross-reactivity common
Infectious virus	Acute phase (DPO 0–7)	Virus isolation, plaque assay, focus-forming assay, TCID_50_	Reference virology, virus characterization, infectivity assessment	Requires viable viruses, biosafety facilities and cell culture; time-consuming
Mosquito viral RNA	During virus circulation in vector populations	RT-qPCR, RT-LAMP, RT-RPA, portable PCR, sequencing	Vector surveillance, early warning, transmission mapping	Pooling dilution, mosquito-derived inhibitors, RNA degradation and complex field sample processing

**Table 2:** Specimen types for MBV detection and their methodological considerations.

Specimen Type	Main Use	Suitable Methods	Advantages	Main Limitations
Serum	Acute diagnosis, serology, reference testing	RT-qPCR, RT-LAMP, NS1 ELISA, IgM/IgG ELISA, PRNT	Well-characterized; compatible with most reference assays	Requires venipuncture, centrifugation and cold-chain storage
Plasma	Acute molecular diagnosis, antigen testing	RT-qPCR, RT-LAMP, antigen assays, serology	Like serum; useful for RNA and antigen detection	Anticoagulants and processing conditions may affect assays
Whole blood	Point-of-care and field diagnosis	Direct RT-LAMP, RT-RPA, portable PCR, LFA	Finger-prick compatible; minimal processing	Contains amplification inhibitors; matrix-specific validation required
FTA card	Remote sampling, transport, surveillance	RT-PCR, RT-qPCR, serology	Improved transport stability; reduced cold-chain need	Lower sample volume and recovery; sensitivity may decrease
Urine	Complementary diagnosis, especially for ZIKV	RT-qPCR, RT-LAMP, CRISPR-based assays	Non-invasive; may extend detection window for ZIKV	Variable and often low viral RNA; possible inhibitors
Saliva	Non-invasive screening or surveillance	RT-qPCR, RT-LAMP, antigen or antibody assays	Easy collection; suitable for repeated sampling	Low and variable viral load; nucleases and mucins may interfere
CSF	Neuroinvasive disease diagnosis	IgM ELISA, RT-qPCR, sequencing	Important for JEV, WNV and neurological cases	Invasive collection; not suitable for field screening
Semen	ZIKV-specific investigation	RT-qPCR, virus isolation in selected studies	Relevant for prolonged ZIKV RNA detection and sexual transmission	Complex matrix; RNA positivity does not necessarily indicate infectious virus
Individual mosquitoes	Vector infection studies, high-resolution surveillance	RT-qPCR, RT-LAMP, sequencing	Avoid dilution; species-specific information	Labor-intensive and costly for large-scale surveillance
Mosquito pools	Routine vector surveillance	RT-qPCR, RT-LAMP, RT-RPA, portable PCR	High throughput; cost-effective	Pooling dilution; requires careful interpretation of infection rates
Mosquito homogenates	Molecular or antigen testing of vectors	RT-qPCR, RT-LAMP, CRISPR, biosensors	Important for vector-derived specimens	Inhibitors, RNA degradation and variable homogenization efficiency
Sentinel animal specimens	One Health surveillance	RT-qPCR, ELISA, PRNT, sequencing	Early warning for enzootic transmission	Requires host-specific validation and ecological interpretation
Environmental samples	Complementary surveillance	RT-qPCR, sequencing	Potential population-level monitoring	Limited evidence for many MBVs; concentration and inhibition challenges

**Table 4:** Sample processing strategies.

Sample	Processing Method	Suitable Methods	Advantage	Limitation	Field Suitability
Serum/plasma	extraction kit	RT-qPCR/CRISPR	clean RNA	equipment needed	medium
Whole blood	direct lysis	RT-LAMP/RPA	simple	inhibitors	high if validated
Urine	concentration/extraction	NAAT	non-invasive	low viral load	medium
Mosquito pool	homogenization + extraction	RT-qPCR/LAMP	surveillance	inhibitors, pooling dilution	medium
DBS/FTA card	dried storage	PCR/LAMP	transport stable	lower recovery	high

## Data Availability

No new data were created or analyzed in this study. Data sharing is not applicable to this article.
